# Gimbal Influence on the Stability of Exterior Orientation Parameters of UAV Acquired Images

**DOI:** 10.3390/s17020401

**Published:** 2017-02-18

**Authors:** Mateo Gašparović, Luka Jurjević

**Affiliations:** Chair of Photogrammetry and Remote Sensing, Faculty of Geodesy, University of Zagreb, Zagreb 10000, Croatia; ljurjevic@geof.hr

**Keywords:** gimbal, exterior orientation parameters, photogrammetry, Inertial Measurement Unit (IMU), Unmanned Aerial Vehicle (UAV)

## Abstract

In this paper, results from the analysis of the gimbal impact on the determination of the camera exterior orientation parameters of an Unmanned Aerial Vehicle (UAV) are presented and interpreted. Additionally, a new approach and methodology for testing the influence of gimbals on the exterior orientation parameters of UAV acquired images is presented. The main motive of this study is to examine the possibility of obtaining better geometry and favorable spatial bundles of rays of images in UAV photogrammetric surveying. The subject is a 3-axis brushless gimbal based on a controller board (Storm32). Only two gimbal axes are taken into consideration: roll and pitch axes. Testing was done in a flight simulation, and in indoor and outdoor flight mode, to analyze the Inertial Measurement Unit (IMU) and photogrammetric data. Within these tests the change of the exterior orientation parameters without the use of a gimbal is determined, as well as the potential accuracy of the stabilization with the use of a gimbal. The results show that using a gimbal has huge potential. Significantly, smaller discrepancies between data are noticed when a gimbal is used in flight simulation mode, even four times smaller than in other test modes. In this test the potential accuracy of a low budget gimbal for application in real conditions is determined.

## 1. Introduction

Unmanned Aerial Vehicle (UAV) photogrammetry has been advancing at a fast pace in recent years. This is primarily the result of the development of Micro Electro Mechanical Systems and Nano Electro Mechanical Systems sensors [1], whose performances have improved several dozen times over the last two decades, while computers, batteries, and cameras are the main limiting factors of UAV photogrammetry. Based on the previous statement, a gimbal soon became a mandatory part of UAV equipment. A gimbal can be used both with fixed wing [2] and multirotor UAVs [3], even though the vibration influence is not significant in fixed wing application and is not usually necessary to use. A gimbal smooths the angular movements of a camera and provides advantages for acquiring better images; this subject and the importance of using and testing gimbals on UAVs is discussed by various authors [4,5]. Additionally, a gimbal dampens vibrations, which is significantly beneficial for real time image stabilization applications [6]. Apart from smoothing angular movements and dampening vibrations, a gimbal maintains a camera in a predefined position. This is mostly the position in which the camera’s axis is horizontal or vertical, but all other positions according to the gimbal’s technical capabilities are possible. This very fact has encouraged interested into the research of gimbal capabilities in maintaining angular parameters of exterior orientation. The main interest is focused on the current capabilities of low budget gimbals, and therefore both potential and possible technological applications, not only in geodesy, but also in other professions, can be foreseen [3,7,8,9]. The primary potential of this technology in geodesy is seen in photogrammetry application without the use of Ground Control Points (GCP) [10,11] and in aerial photogrammetry simulation, for maintaining a parallel camera axis (i.e., normal case). Photogrammetry without the use of GCP requires known exterior orientation parameters. Furthermore, there are different engineering applications, for example high accuracy stakeouts without the use of a total station, and so forth.

Considering that gimbals are nothing new in photogrammetry, one would expect this topic to be well researched. However, most authors are not researching UAV gimbals in this way. Most of the papers are based on the use of gimbal or exterior orientation parameters determination through Global Navigation Satellite System (GNSS) and Inertial Navigation System (INS) integration [12,13]. Also, most of the papers are based on determining only the spatial parameters of the exterior orientation [14]. In these papers, authors commonly use expensive gimbals with sophisticated IMUs. However, in this paper the impact of a low budget gimbal on the determination of only part of the rotational parameters of the exterior orientation will be researched using photogrammetric and IMU acquired data. Only roll and pitch (ω,φ) will be researched; yaw (κ) will not be the subject of this research because in photogrammetry yaw is mostly referred to the UAV, and not absolutely from the reference coordinate system.

## 2. Technology

Regarding the UAV photogrammetry, two types of UAVs are used: fixed wing and multirotor. Multirotor UAVs are characterized by better maneuverability and generally have better accuracy of object reconstruction because they are able to approach closer to the object. On the other hand, fixed wing UAVs are characterized by larger area coverage and longer flight time. The flight of fixed wing UAVs is generally more stable, so gimbals are rarely used on fixed wing UAVs. However, a camera system on a multirotor UAV is exposed to motor vibrations and sudden altitude changes, so it is recommended to use a gimbal in order to assure quality cadre, endlap, sidelap, and the acquisition of sharp images. In this paper the exterior orientation stability is analyzed, which is directly transferred to an image perspective, respectively, quality cadre, endlap, and sidelap. An upgraded multirotor UAV Cheerson CX-20 Open Source was used in this paper. The upgrades done on the UAV include mounting a gimbal and connecting it to a flight controller, extending the landing gears, using advanced GNSS antenna and a bigger capacity battery, and mounting new and better propellers.

One of the limiting factors of UAVs is the weight of the payload, and the main payload of the UAV is the camera. The maximal payload depends on the UAV’s parameters, motor power, and battery capacity. In the past, it was mandatory to use metric cameras, but nowadays the situation is very different because the manufacturing quality of digital cameras has improved. Considering this, it is advised to assume the need to use a metric camera, because an amateur camera would not be sufficient. The greatest impact on the accuracy of a photogrammetric survey based on amateur cameras is lens distortion [15]. Cameras are differentiated by various parameters, but for photogrammetric applications the most important parameters are optics quality, sensor quality, and lens compatibility [16].

**Figure 1:**
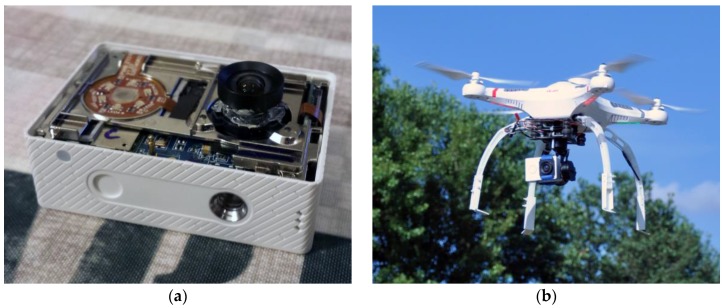
A Xiaomi Yi action camera; (**a**) in improving phase; (**b**) on a 3-axes gimbal on an Unmanned Aerial Vehicle (UAV).

In order to use a camera in photogrammetric applications it is necessary to know its interior orientation parameters. Interior orientation is determined by camera calibration. For the purposes of this paper, the calibration of an improved camera, Xiaomi Yi, with declared low distortion lens and focal length of approximately 4.35 mm, was done in Orpheus (version 3.2.1). A Xiaomi Yi is a low budget action camera with a fixed lens. The specifications of the improved Xiaomi Yi camera are specified in Table 1, and both uses of the camera and the UAV are shown in Figure 1a,b.

The Xiaomi Yi camera was exposed to software enhancement by scripts that were developed for storing the images taken in raw + JPG (100% quality) format. The Xiaomi Yi camera by default only has the capability to store filtrated JPG images of limited quality. The post-processed raw format potentially allows access to better quality images than the original JPG images. Furthermore, a rolling shutter effect was encountered and eliminated with a combination of appropriate illumination, reduced exposure time to 1/1002 s (fixed value), and lower flight velocity. The exposure time was manipulated via scripts development.

In the UAV domain, a gimbal is a device that maintains orientation and dampens vibrations. The task of a gimbal is to calculate a correction for every detected movement in the unit of time *i*, and compensate for it while meeting the following requirement:

(1) $$\omega_{T}\approx \omega_{i},\varphi_{T}\approx \varphi_{i},\kappa_{T}\approx \kappa_{i}$$

$\omega_{T},\varphi_{T},\kappa_{T}$ are predefined rotation angles for each gimbal axis. Unlike angles $\omega_{T},\varphi_{T}$, angle $\kappa_{T}$ is not absolutely defined by the direction of gravity, but relatively to the UAV or absolutely in reference to the direction north. The frequency of the gimbal is 700 Hz, which means that the correction rate is calculated using the following expression:

(2) 700Hz⇒i=1/700s

At the same time, the gimbal forms the connection between the UAV and the camera system. Depending on the number of gimbal axes, it stabilizes the camera along two or three axes. These are pitch and roll (φ,ω), or pitch, roll, and yaw (φ,ω,κ) axes. The gimbal functionality is controlled by a gimbal controller which calculates corrections for the camera position based on the horizon deflection of the IMU mounted on the camera holder. 

**Figure 2:**
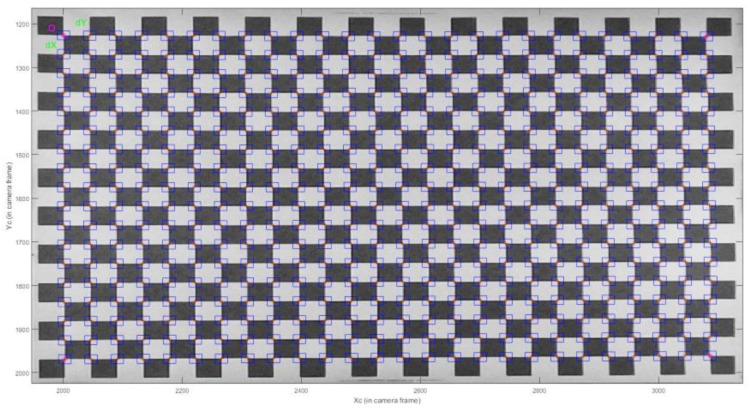
Chessboard test field Ground Control Point (GCP) detection in Matlab.

For the purposes of flight simulation and indoor flight tests, a chessboard test field of the dimensions 81 × 57 cm, with a square size of 3 cm, has been created. During the flight simulation and indoor flight test the chessboard test field was continuously shot. Exterior orientation parameters were calculated in Orpheus software based on the images taken. A detailed description of the test procedure is described in Section 4. Part of the Matlab toolbox implemented in C and included in OpenCV was used for the chessboard’s GCP detection [17]. Automatic GCP detection was the subject of research of numerous authors apart from Jean-Yves Bouguet [18,19]. Automatic detection software was used in order to save time and to eliminate the influence of operator errors. In order to import data into the Orpheus software, a Matlab (version 9.0) script for data preparation has been created. Figure 2 represents a visualization of the chessboard test field GCP detection. The exterior orientation parameters for outdoor flight were calculated in Agisoft PhotoScan software (version 1.2.6, 64 bit).

## 3. Methodology

The main goal of this study is to examine the possibility of obtaining better geometry and favorable spatial rays of bundles of images, and respectively better image cadre, endlap, and sidelap in the UAV photogrammetric survey. The research methodology and a detailed description, with an explanation of the mathematical models used in order to carry out the tests and design a new method, is given in this section. In order to explain how the analyzed data are collected, it is necessary to discuss the photogrammetric methods that were used and the IMU operating principles, which are presented in Section 3.1 and Section 3.2.

### 3.1. Photogrammetric Mathematical Models

**Figure 3:**
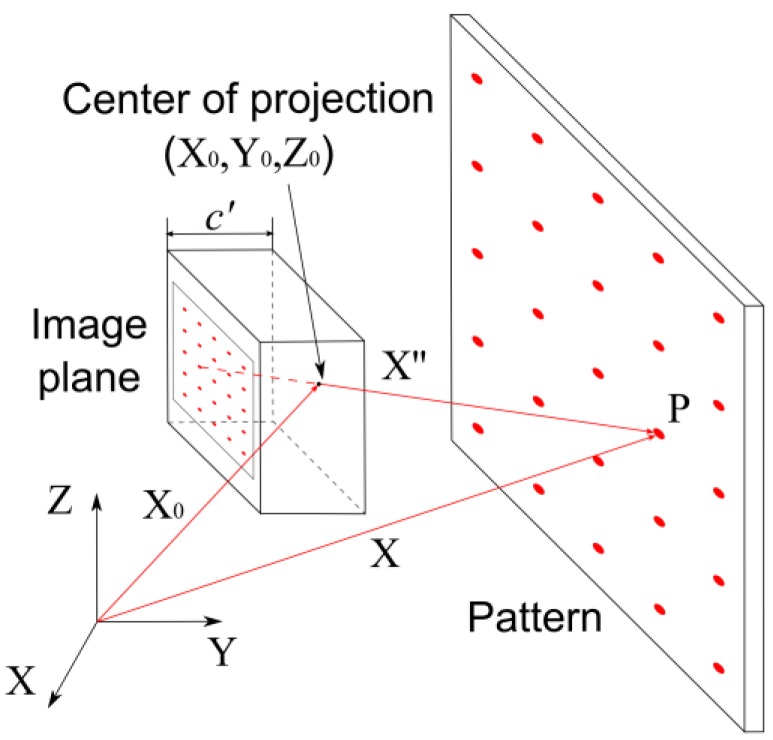
Pinhole camera model.

The pinhole camera model is the most frequently used mathematical model of a camera in photogrammetry (Figure 3). It is a model of projection where a light ray passes from every point on an object through the center of a projection and ends in the image plane. According to this mathematical model, the point on the object, the center of the projection, and the point in the image plane are collinear.

The center of the projection is defined by vector X_0_ in reference to the coordinate system. Point P (vector X), located on object, can be derived based on vector X_0_ and vector X″, according to Equation (3). Vector X″ is defined with the point on the object and the center of projection.

(3) $$X=X_{0}+X^{\prime \prime }$$

If only one image of the given object is available, only the direction of point P can be defined. In order to determine the coordinates of point P, at least one more image of the given object from a different position is required. This kind of projection is described with collinearity equations [20].

(4) $$\begin{array}{l} x^{\prime }={x^{\prime }}_{0}-c^{\prime }\frac{r_{11}\cdot \left(X-X_{0}\right)+r_{21}\cdot \left(Y-Y_{0}\right)+r_{31}\cdot \left(Z-Z_{0}\right)}{r_{13}\cdot \left(X-X_{0}\right)+r_{23}\cdot \left(Y-Y_{0}\right)+r_{33}\cdot \left(Z-Z_{0}\right)}+\Delta x^{\prime },\\ y^{\prime }={y^{\prime }}_{0}-c^{\prime }\cdot \frac{r_{12}\cdot \left(X-X_{0}\right)+r_{22}\cdot \left(Y-Y_{0}\right)+r_{32}\cdot \left(Z-Z_{0}\right)}{r_{13}\cdot \left(X-X_{0}\right)+r_{23}\cdot \left(Y-Y_{0}\right)+r_{33}\cdot \left(Z-Z_{0}\right)}+\Delta y^{\prime }, \end{array}$$

where *x*′, *y*′ are the point P image coordinates, *x*_0_′, *y*_0_′ are the Principal Point of Autocollimation (PPA) image coordinates, *c*′ is the camera constant, *r_ij_* is the spatial rotational matrix parameter, *X*, *Y*, *Z* are point P coordinates in reference to the coordinate system, *X*_0_, *Y*_0_, *Z*_0_ are the projection center coordinates in reference to the coordinate system, and ∆*x*′, ∆*y*′ are the distortion parameters.

Collinearity equations express image coordinates (*x*′, *y*′) as a function of the interior orientation (*x*_0_′, *y*_0_′, *c*′, ∆*x*′, ∆*y*′) and the exterior orientation (*X*_0_, *Y*_0_, *Z*_0_, φ, ω, κ) parameters of a single image. Bundle Block Adjustment (BBA) is applied when adjusting interior and exterior orientation parameters for an arbitrary number of images, which are connected in a single 3D model. Within BBA, observations (image coordinates), classic survey measurements (GCP), and the referent coordinates of the object are adjusted simultaneously. Equation (5) represents the indirect measurements function model.

(5) $$\begin{array}{l} x\prime_{i}+vx\prime_{i}=F\left(\bar{X_{oj},Y_{oj},Z_{oj},\omega_{j},\varphi_{j},\kappa_{j},x\prime_{0k},c_{k},\Delta x\prime_{k},X_{i},Y_{i},Z_{i}}\right),\\ y\prime_{i}+vy\prime_{i}=F\left(\bar{X_{oj},Y_{oj},Z_{oj},\omega_{j},\varphi_{j},\kappa_{j},y\prime_{0k},c_{k},\Delta y\prime_{k},X_{i},Y_{i},Z_{i}}\right)\;, \end{array}$$

where *i* is the point of index, *j* is the image index, and *k* is the camera index.

The approximate unknowns of the exterior orientation are determined analytically out of at least three known non-collinear points on the object. Images are tied up together with different image matching techniques [21] (the automatic detection of tie points) or by the observation of GCP. A combination of image matching and GCP observation is possible if the GCP are not visible on every image. Images are oriented mutually by intersecting all corresponding rays (i.e., homologous rays). This way, a strong geometry is created. Interior orientation (*x*_0_′, *y*_0_′, *c*′, ∆*x*′, ∆*y*′), exterior orientation (*X*_0*j*_, *Y*_0*j*_, *Z_0j_*, $\varphi_{j},\omega_{j},\kappa_{j}$), and object points (*X_i_*, *Y_i_*, *Z_i_*) in reference to the coordinate system are determined in a single adjustment. The statements above indicate that BBA is mathematically the most acceptable method of image orientation in the domain of photogrammetry. 

The approach described above is used in an outdoor flight test. In flight simulation and indoor flight exterior orientation determination a similar approach was used, only the interior orientation parameters were not adjusted. Therefore, the interior orientation parameters were predetermined with the camera calibration and entered the exterior orientation adjustment (space resection) as fixed values. The Xiaomi Yi camera was calibrated with a test field calibration method in Orpheus software on a test field with 468 points. The calibration algorithm is explained by Gašparović and Gajski [22]. Agisoft PhotoScan software was used in order to determine the exterior orientation parameters during the outdoor flight due to its ability to automatically determine a large number of tie points that enter adjustment and consequently improve the quality of the external orientation parameters. Brown [23] and TU Wien [24] distortion models were used in this paper.

### 3.2. IMU Data Integration

**Figure 4:**
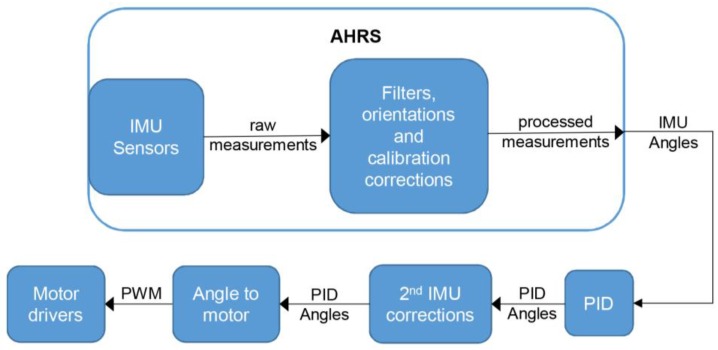
Control loop of the gimbal controller.

The primary gimbal IMU consists of a 3-axis accelerometer and a 3-axis gyroscope, and it is mounted onto the gimbal’s camera holder. The gimbal’s control loop is shown in Figure 4. The raw measurements are processed with filters, calibration, and orientation corrections. Usually a Kalman filter is used [25]. The Attitude Heading and Reference System (AHRS) calculates the orientation angles based on the corrected IMU measurements. Based on the AHRS data, the PID (Proportional Integral Derivative) angles are calculated by a PID controller and sent via Pulse-Width Modulation (PWM) to a motor driver, which is a moving camera, to correct the position.

Besides the primary IMU, the gimbal can use another, secondary IMU that is located on the controller board. The secondary IMU has to be mounted independently of any motor. The use of a secondary IMU is remarkably beneficial because it contributes to a significant accuracy and increase in operational range [26], as well as maintaining a more stable yaw axis.

Various IMUs based on cheap Micro Electro Mechanical System sensors are available on the market and almost every smartphone is equipped with a cheap IMU. They are subject to systematic influence errors due to imprecise scaling factors [27] and non-perpendicular axes, which results in a reduced accuracy of its position and direction [28].

In order to reduce the aforementioned errors, the IMU should be calibrated. IMU calibration is a crucial step when ensuring the gimbal’s optimal performance. If both the primary and the secondary IMU are used, they should be calibrated. For the purposes of this paper, a 1-point calibration method was used. A 1-point calibration method was conducted by stabilizing the IMU on the horizontal plane and measuring the IMU accelerometer data while it was completely still. During calibration, it is important to take into account exterior influences that are causing vibrations. The ambient temperature at which the calibration is done should be as close as possible to the ambient temperature in which the gimbal will be used.

**Figure 5:**
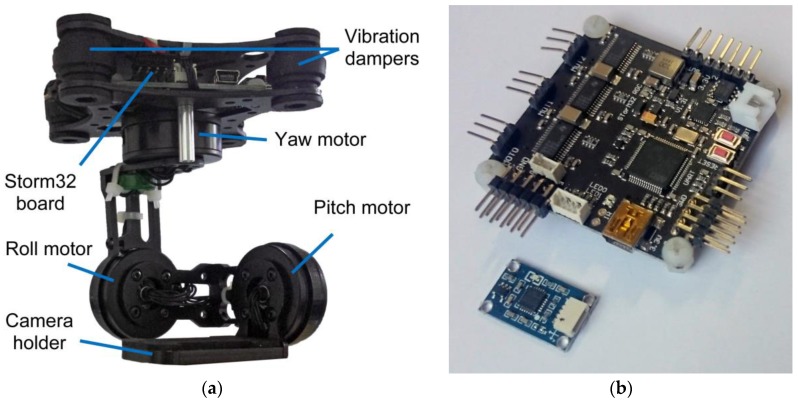
(**a**) Used gimbal; (**b**) gimbal controller (Storm32) board and primary Inertial Measurement Unit (IMU) (MPU6050).

The IMU was stabilized on an autograph WILD A7 image insertion plane, whose horizontality was tested with a calibration level of 3” sensitivity. The primary and secondary IMUs were mounted to the horizontal plane and were left for some time while their measurements were stabilized. The calibration was performed based on the measurements after they became stabilized, while the IMU measurements were logged and analyzed in order to control the IMU. The IMU measurements can be logged as long as the gimbal controller is connected via a USB to the computer. In this paper, a gimbal controller Storm32 (Olliw.eu, Denzlingen, Germany) with a primary and integrated secondary IMU MPU6050 (InvenSense, San Jose, USA) was used. The integration between these two IMUs are based on the I2C communication protocol. Figure 5a represents the gimbal used and Figure 5b represents the gimbal controller and the primary IMU that were used.

The used gimbal is based on two IMUs for the calculation of horizon deflection and correction. During the research a drift along the roll axis was noticed and it was approximately 1° per minute. The noticed drift was almost totally eliminated by adjusting the IMU AHRS parameter, which controls the coefficient of the accelerometer and the gyroscope data when entering the AHRS algorithm. Significant improvement was achieved by setting the IMU AHRS parameter so that the algorithm uses only accelerometer data. This is probably a consequence of the inability of the software used to calibrate the IMU’s gyroscopes. 

Other than that which has been mentioned, the measurements and usage of the IMU data were controlled by Gyro Low Pass Filter and IMU 2 Feed Forward Low Pass Filter parameters. The Low Pass Filter passes data with a frequency lower than the one that is set, and attenuates data with higher frequency. The Gyro Low Pass Filter parameter is used to set the aforementioned frequency for the gyroscope data. This process is used in order to filter the vibration from the real data. The IMU 2 Feed Forward Low Pass Filter is also used to set the Low Pass Filter frequency for the attitude data before entering the Feed Forward channel.

## 4. Experimental Approach

The exterior orientation stability was tested in three different environments:

- Flight simulation,
- Indoor flight,
- Outdoor flight.

The main goal of this experiment is to determine the improvement of exterior orientation stability using a gimbal, and to define a new method for testing gimbal stability. In all three tests the Xiaomi Yi camera with identical predetermined internal orientation parameters was used. In order to ensure the quality of the research, all tests were shot in more than one session. While the camera was mounted onto the UAV without a gimbal, its attitude was directly correlated to the rotational parameters of the camera’s exterior orientation. Considering this, the UAV body movement is logged as secondary IMU data for the flight simulation test due to having an achievable cable connection with a computer, while for indoor as well as outdoor test flights the UAV body movement was logged as flight controller IMU data. This data also represents the camera’s exterior orientation parameters without the use of the gimbal. The UAV’s flight controller is APM 2.52 (ArduPilot, Indianapolis, USA), which uses onboard MPU6000 IMU (InvenSense, San Jose, USA). The used Storm32 gimbal controller has the capability to log both of the IMUs’ data and these data is used to analyze the exterior orientation parameters. In the case of an ideal gimbal, the exterior orientation parameters would not change during the time. The analyzed data is acquired in three different tests, with flight simulation, indoor flight, and outdoor flight. During the tests, it was noticed that the gimbal’s initial position changes by a small angular value every time it is powered, but this fact is noted and ignored because the subject of this paper is stability analysis, which this change does not influence. Therefore, the photogrammetrical data presented is normalized in order to minimize the influence of difference in the initializations for each session.

### 4.1. Flight Simulation

Flight simulation was taken in front of the chessboard test field that contains 468 GCP in total. During the flight simulation, the UAV was turned on in order to power the gimbal, but the UAV motors were disabled. The movement of the UAV is simulated manually by rotating it up to a maximum of ±45° across the roll and pitch axes. Analysis was carried out on the secondary IMU data during the 15 min of flight simulation. In addition to the IMU data, the exterior orientation stability was analyzed via data acquired with the photogrammetric method. During the 15 min of flight simulation, 36 images were taken and the secondary IMU data was logged. The camera and IMU are synchronized manually with sufficient precision for this type of test by starting to record the IMU data when the camera signals the start of the time lapse data acquisition. The time lapse interval was set to 25 s. It is worth noting that data synchronization is not subject of this paper and that the analysis was done with reference to a range of data. The flight simulation was done in order to log the gimbal IMU data, which was not possible during the real flight due to the requirement of a cable connection between the gimbal controller and the computer. The exterior orientation parameters were calculated for every image with Orpheus software on the test field with 468 GCP, as it is described in Section 3.1.

### 4.2. Indoor Flight

**Figure 6:**
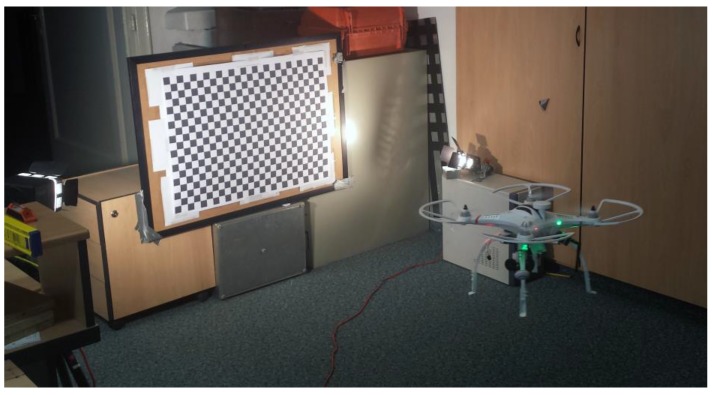
Indoor flight test.

After analyzing the exterior orientation stability during the simulated indoor flight, real flight testing was conducted. The flight was performed in front of the already mentioned test field with 468 GCP, in an indoor environment. Three sessions were carried out and the duration of them was 15 min, in which 204 test field images were taken, approximately one image for every four seconds. Images and IMU data synchronization is done by coincidencing the system time of the camera and flight controller. For data analysis, only images taken inside an imaginary sphere with a radius of 0.5 m were chosen. The center of the imaginary sphere is the average value of the exterior orientation spatial parameter out of all 204 images taken. It was done in order to exclude all images that were taken too close or too far from the test field due to the demands of controlling the UAV indoors. For the purpose of smoother indoor flight, the UAV maximal pitch and roll tilt was limited to ±15° via the flight controller angle maximal value. Figure 6 shows the UAV during the indoor flight test.

In total, 137 images were used, which makes 67% out of all 204 images. As described in the above sections, the exterior orientation parameters were calculated with Orpheus software on the test field with 468 GCP for every image. The flight controller IMU data was used to test the exterior orientation accuracy improvement.

### 4.3. Outdoor Flight

Outdoor flight was carried out in two sessions. The test area was a rocky terrain shot with a camera axis directed vertically to the ground. The UAV was controlled by an autonomous mission through a predefined trajectory in both sessions. This makes outdoor flight much smoother and more stable than indoor flight. Because the UAV is influenced by weather conditions (e.g., wind), the maximal pitch and roll tilt value is higher than it is in indoor flight, and in this outdoor flight test it was limited to ±25°. The first session was carried out on the relative height of 40 m above the area of 4.5 ha, with 80% endlap and 60% sidelap, while the second session was carried out on the relative height in the range from 50 m to 70 m above of 6 ha, with 85% endlap and 50% sidelap. In total, 5 GCP were regularly distributed over the subject area, and 13211 tie points were used to align the first session of data, and to align the second session data 7 GCP regularly distributed over the subject area and 18,324 tie points were used. The flight velocity was 4 m/s in both sessions. During the first session 191 images were shot, and during the second session 141 images were shot, approximately one image for every two seconds for both sessions. The images and IMU data synchronization is done by coinciding the system time of the camera and the flight controller. Images on the ends of the strips were not taken into consideration because the exterior orientation data of those images would corrupt the statistical data, and they do not represent real values because on the ends of the strip the terrain is afforested and lacks the GCP and tie points necessary for quality orientation. Additionally, the turns from one strip to another are the most challenging maneuvers for a gimbal to compensate. The analysis was carried out on 47 images from the first session and 51 images from the second session, as in the flight controller IMU data. The exterior orientation parameters were calculated with Agisoft PhotoScan software because it can automatically determine a large number of tie points that enter adjustment.

## 5. Results

The results of the tests taken in order to analyze the impact of the gimbal on the stability of exterior orientation, and therefore its ability to achieve a better geometry of bundles of rays in UAV photogrammetry, which are explained in the above section, will be represented here.

### 5.1. Flight Simulation

Presented in Table 2 are the statistical data acquired photogrammetrically and by the secondary IMU, which represents the movement of the camera in use without the gimbal.

**Figure 7:**
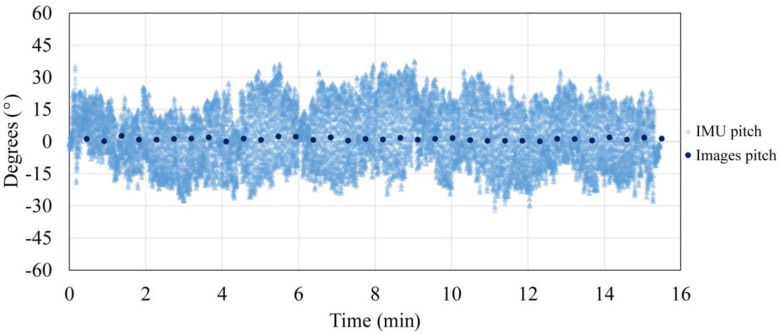
Exterior orientation parameters acquired photogrammetrically and by the Inertial Measurement Unit (IMU) for pitch parameter.

**Figure 8:**
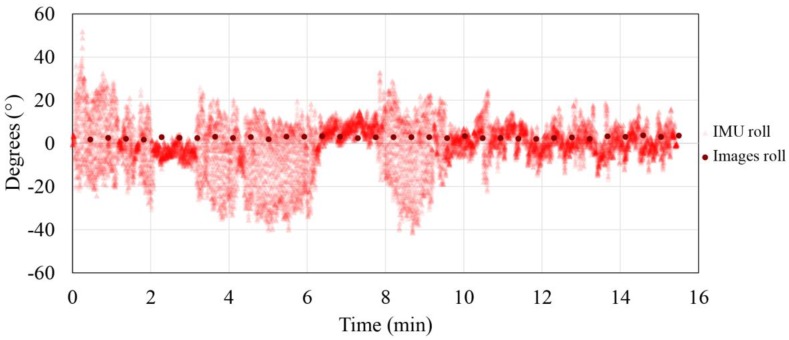
Exterior orientation parameters acquired photogrammetrically and by the Inertial Measurement Unit (IMU) for roll parameter.

The photogrammetrically and secondary IMU acquired data ratio for roll and pitch parameters are represented in Figure 7 and Figure 8. Photogrammetrically acquired data are represented in reference to the arithmetic mean (normalized data) in order to eliminate the influence of the gimbal initialization and the non-verticality of the test field.

From the presented data, improvement of the exterior orientation stability as a consequence of gimbal use can be seen. The roll and pitch parameters determined independently with a photogrammetric method (the camera is stabilized with a gimbal) are between 2.56° and 1.97°, with a standard deviation from 0.46° and 0.36°, while the secondary IMU (platform instability) data range from 69.90° and 93.18°. From the previous data value, the advantage of using a gimbal is evident. In addition, it is shown that the pitch parameter statistic is slightly worse than the roll parameter statistic, which can be explained as a consequence of the planar test field usage, which benefits roll parameter determination in opposition to the 3D test field, which benefits pitch parameter determination.

### 5.2. Indoor Flight

Statistical data acquired photogrammetrically using a gimbal is presented in Table 3.

**Figure 9:**
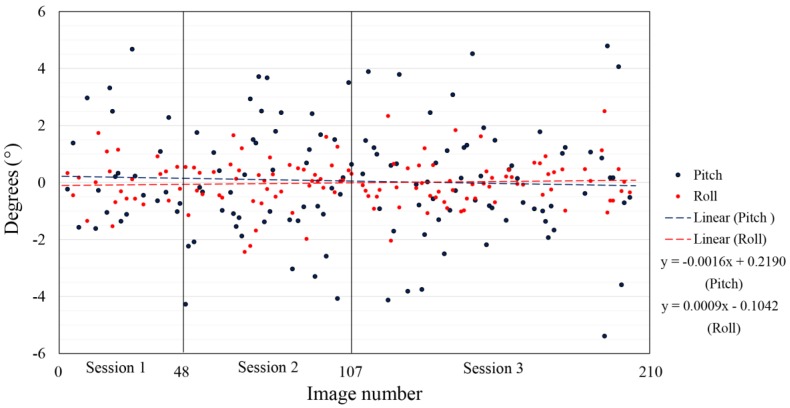
Roll and pitch parameter values and trend line for three sessions of indoor flight.

The exterior orientation parameters calculated for three sessions of indoor flight, all in reference to the arithmetic mean for every session (normalized data) and trend line for the roll and pitch parameters, are represented in Figure 9. In this visualization, the gimbal initialization is taken into account. After every start-up, the gimbal is initialized and in this visualization the difference in initializations is disregarded. In Figure 9 the repeatability of the data from the three different sessions is shown. In all three sessions, the range value of the roll and pitch measurements and the standard deviation is very similar. The arithmetic mean of the third session differs from the arithmetic mean of the first and second sessions, which can be interpreted as a significantly different initialization of the gimbal. Due to specific flight conditions (indoor flight), the flight controller was limited to a ±15° tilt, which resulted in three times improved stability of the exterior orientation with the use of a gimbal compared to without it.

### 5.3. Outdoor Flight

**Figure 10:**
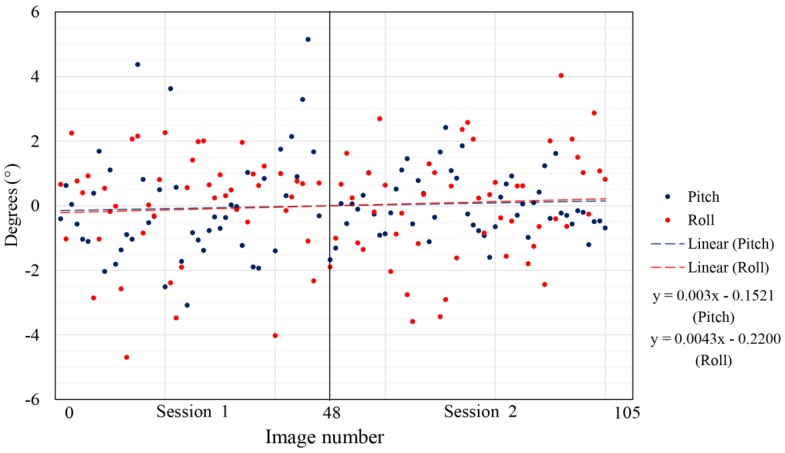
Roll and pitch parameter values and trend line for both sessions of outdoor flight.

Data acquired during the two sessions of outdoor flight, both in reference to the arithmetic mean (normalized data) and trend lines of every session, are represented in Figure 10.

The statistical data of the measurements visualized in Figure 10 are represented in Table 4, in which the repeatability of the data from the two different sessions is shown. The only significant difference in the data is a better standard deviation of pitch parameter for the second session compared to the first session. This is very likely to be a consequence of lower flight and the determination of better automatic tie points, in opposition to the higher flight of the first session. For this flight, the flight controller was limited to a ±25° tilt as a standard value for outdoor flights due to external influences, which resulted in six times improved stability of the exterior orientation with the use of a gimbal compared to without it.

## 6. Discussion

The analyzed data were acquired during three different tests, by flight simulation, and indoor and outdoor flight. The range of the minimal and maximal values of roll and pitch parameters calculated photogrammetrically for flight simulation is 2.56° and 1.97°, with a standard deviation of 0.46° and 0.36°. On the other hand, the data acquired during the indoor flight ranges between 8.95°, 10.40° and 10.18°, with a standard deviation of 1.99°, 2.14° and 2.04° for pitch parameter trough sessions, and for the roll parameter data range between 3.27°, 4.09° and 4.54° with a standard deviation of 0.81°, 0.91° and 0.82°. The data acquired during the two sessions of outdoor flight range between 8.23° and 4.09° with a standard deviation of 1.73° and 0.92° for pitch parameter, and the roll parameter data range between 6.65° to 7.61° with a standard deviation of 1.66° and 1.69°. The improvement achieved due to the use of a gimbal depends on the flight conditions, which follows that the level of improvement achieved during the flight simulation is over 20 times, during indoor flight is three times and during outdoor flight is six times compared to the exterior orientation stability without the use of a gimbal. Note that during the indoor and outdoor test flights, the controller was limited to a ±15° and a ±25° tilt, and that during the flight simulation there were no active restrictions. From previously presented results, it is clear that a gimbal improves the exterior orientation stability of the camera. Therefore, using a gimbal on UAVs contributes to a better geometry of bundles of rays, better sidelap and endlap, and better image cadre.

**Figure 11:**
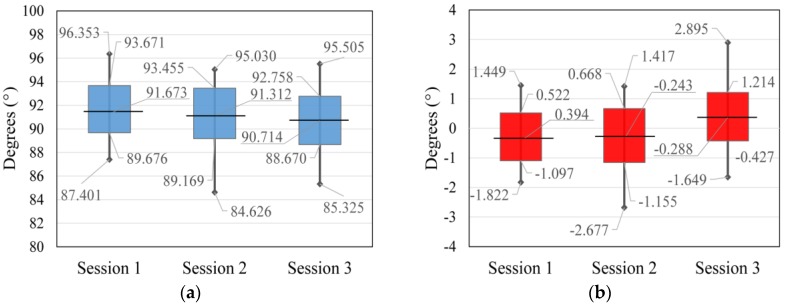
Display of statistical data of indoor flight for: (**a**) pitch parameter and (**b**) roll parameter.

**Figure 12:**
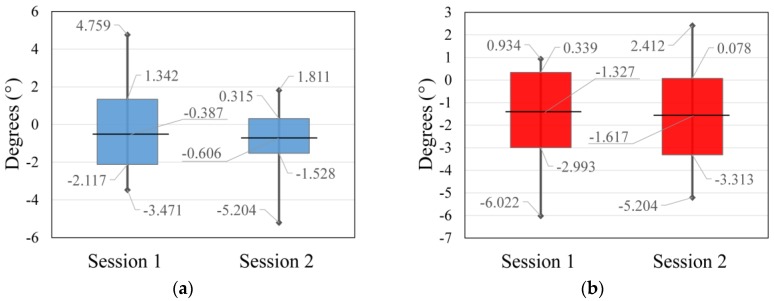
Display of statistical data of outdoor flight for: (**a**) pitch parameter and (**b**) roll parameter.

Data from Table 3 and Table 4 are represented in Figure 11 and Figure 12. The horizontal line defines the arithmetic mean of the session data, the vertical line defines the minimal and maximal measured value of the session, and the top and bottom of the rectangle define 1σ area for every single session.

The data acquired during the flight simulation proved to be the best. The range of the data is smaller and the standard deviation is better than in other tests. This is a consequence of unreal conditions, and almost no shift in the spatial parameters of the exterior orientation and lower vibrations. Regardless, a huge improvement in the exterior orientation stability occurs when using a gimbal. The gimbal’s secondary IMU and the flight controller IMU give us an insight. In contrast, data acquired during the indoor flight were slightly worse due to the nature of flying indoors. The indoor flight was totally manual, a GNSS lock and position hold were not available, and the spatial parameters of the exterior orientation were rapidly and almost randomly changing, which influenced the angular exterior orientation parameters due to correlation. Perhaps the greatest influence on the exterior orientation determination was caused by the test field, which did not fill the whole image cadre, and the consequence is four times worse results for the pitch parameter and two times worse for the roll parameter of the indoor flight in comparison to the flight simulation. In order to confirm the obtained results, an outdoor flight test was taken. The statistics of the pitch parameter of the second session were better than the statistics of the first session. On the other hand, the roll parameter statistics of the second session are slightly worse than the statistics of the first session. The better pitch statistics for the second session is a consequence of a lower flight height in comparison to the first session. Due to the lower flight height, which caused larger perspective differences in the adjacent images, and consequently worse tie point determination, the second session’s roll statistics are slightly worse than first session’s roll statistics. It is important to be aware of small error influences due to the impossibility of matching the projection center and the gimbal axis intersections [29].

## 7. Conclusions

A new approach to exterior orientation stability research using a gimbal is presented in this paper. The new method consists of photogrammetric methods and therefore gives us an external evaluation of stability, in contrast to former research papers on the external orientation of cameras mounted on a gimbal which are based on the IMU data, and only gives us an insight into the internal evaluation of the gimbal’s stability. The expected improvement of the exterior orientation directly depends on the flight mode and gimbal quality, but the expected stability ranges 10° for flight. With the flight simulation test, the goal of stabilization in a real environment is to set up the reachable stabilization accuracy. With the aforementioned test, the data range from 2.56° and 1.97°, and a standard deviation from 0.46° and 0.36° for pitch and roll parameters is acquired. The exterior orientation stability is enhanced 3 or 6 times in comparison to the flight tests, depending on the conditions of the flight. Considering the fact that a gimbal is low budget equipment and that used technology is quickly developing, this goal will be reached soon. According to this new method for gimbal testing, it is clear that the gimbal contributes to better stability of the predefined position of the camera (the exterior orientation) and because of this it can be concluded that the gimbal contributes to better geometry of the bundles of rays, better sidelap and endlap, and better image cadre. The gimbal testing method presented here is a key contribution, and is knowledge that is of significant importance for the UAV photogrammetric survey.

The full potential of used technology and its applications is much bigger than just the stabilization of exterior orientation parameters. Technology development can lead to various applications in geodesy and other professions and the most obvious example of this application is the potential stakeout on challenging terrain using a combination of GNSS measurements and a gimbal to define direction.

Because this paper did not research the influence of gimbals on the linear exterior orientation parameters, it is recommended to determine the linear camera shift constants in reference to the GNSS antenna phase center in further research. Furthermore, the impact of gimbals on radiometric characteristics of images is not researched enough, and future research could focus on this problem due to the high gimbal frequency that influences camera radiometric performance.

## Figures and Tables

**Table 1:** Improved Xiaomi Yi camera.

Processor	Ambarella A7LS
Focal length	4.35 mm (distortion < 1%)
Aperture	F2.8
FOV ^1^ (diagonal)	86°
Sensor	Sony Exmor R BSI CMOS 16 MP
Size	6 × 2.1 × 4.2 cm/2.36 × 0.83 × 1.65 inches
Battery	1010 mAh
Weight	72 g
Video	Up to 1080 p 60 fps
Memory	Up to 64 GB SD card
Connectivity	Wi-Fi, Bluetooth 4.0 v, USB, micro HDMI
Raw data	Yes

^1^ FOV—Field of View.

**Table 2:** Statistics of the Inertial Measurement Unit (IMU) and photogrammetry data for the flight simulation.

	Pitch (°)	Roll (°)	IMU Pitch (°)	IMU Roll (°)
Average	91.13797	2.71445	2.72	−0.71
St. dev.	0.46591	0.36282	13.36	10.94
Min	90.03401	1.75086	−32.17	−41.34
Max	92.59373	3.71907	37.73	51.84

**Table 3:** Statistics for all three sessions of indoor flight.

	1st Session		2nd Session		3rd Session	
	Pitch (°)	Roll (°)	Pitch (°)	Roll (°)	Pitch (°)	Roll (°)
Average	91.67314	−0.28787	91.31183	−0.24313	90.71399	0.39360
St. dev.	1.99739	0.80951	2.14277	0.91155	2.04433	0.82016
Min	87.40141	−1.82238	84.62633	−2.67715	85.32468	−1.64857
Max	96.35258	1.44858	95.02984	1.41679	95.50489	2.89549

**Table 4:** Statistics for both sessions of outdoor flight.

	1st Session		2nd Session	
	Pitch (°)	Roll (°)	Pitch (°)	Roll (°)
Average	−0.38750	−1.32690	−0.60653	−1.61740
St. dev.	1.72962	1.66566	0.92112	1.69534
Min	−3.47135	−6.02212	−2.28265	−5.20398
Max	4.75898	0.93433	1.81105	2.41166
